# Iron Deficiency in Heart Failure: Mechanisms and Pathophysiology

**DOI:** 10.3390/jcm11010125

**Published:** 2021-12-27

**Authors:** Ridha I. S. Alnuwaysir, Martijn F. Hoes, Dirk J. van Veldhuisen, Peter van der Meer, Niels Grote Beverborg

**Affiliations:** Department of Cardiology, University Medical Center Groningen, University of Groningen, P.O. Box 30.001, 9700 RB Groningen, The Netherlands; r.i.s.alnuwaysir@umcg.nl (R.I.S.A.); m.hoes@umcg.nl (M.F.H.); d.j.van.veldhuisen@umcg.nl (D.J.v.V.); p.van.der.meer@umcg.nl (P.v.d.M.)

**Keywords:** iron deficiency, iron metabolism, heart failure, pathophysiology

## Abstract

Iron is an essential micronutrient for a myriad of physiological processes in the body beyond erythropoiesis. Iron deficiency (ID) is a common comorbidity in patients with heart failure (HF), with a prevalence reaching up to 59% even in non-anaemic patients. ID impairs exercise capacity, reduces the quality of life, increases hospitalisation rate and mortality risk regardless of anaemia. Intravenously correcting ID has emerged as a promising treatment in HF as it has been shown to alleviate symptoms, improve quality of life and exercise capacity and reduce hospitalisations. However, the pathophysiology of ID in HF remains poorly characterised. Recognition of ID in HF triggered more research with the aim to explain how correcting ID improves HF status as well as the underlying causes of ID in the first place. In the past few years, significant progress has been made in understanding iron homeostasis by characterising the role of the iron-regulating hormone hepcidin, the effects of ID on skeletal and cardiac myocytes, kidneys and the immune system. In this review, we summarise the current knowledge and recent advances in the pathophysiology of ID in heart failure, the deleterious systemic and cellular consequences of ID.

## 1. Introduction

Heart failure (HF) is a complex syndrome in which the heart fails to circulate the required amount of blood and nutrients to meet the body’s demands [1]. Despite substantial advances in prevention and treatment strategies of HF, it continues to represent a huge burden on public health worldwide, with an exceptionally high mortality rate reaching up to 75% at 5 years [2]. This makes HF as ‘malignant’ as some types of cancer [3,4]. Multimorbidity in the ageing population of HF is becoming increasingly frequent, with almost half of the patients presenting with five or more noncardiac comorbidities [4,5]. The presence of comorbidities increases the complexity of treating HF and impairs quality of life and clinical outcomes [6,7]. Therefore, management of comorbidities in addition to the mainstay therapies of HF constitutes a crucial aspect of HF treatment [8].

Iron deficiency (ID) and anaemia are among the most frequently observed comorbidities in HF, and both are independently associated with worse clinical status and outcomes [9,10,11]. Not only the presence of these comorbidities has been associated with worse prognosis, but also the severity, with advancing severity associated with higher mortality rates [10]. Although ID has traditionally been linked with anaemia, commonly referred to as iron deficiency anaemia (IDA), the two conditions do not necessarily coexist [12]. In fact, ID is poorly linked with red cell indices in HF, indicating that ID in these patients should be seen independently of erythropoietic status [13].

ID is substantially more prevalent than anaemia in HF, with a prevalence reaching up to 59% even in non-anaemic ambulatory HF patients [10,14,15,16,17]. In acute HF, the prevalence of ID among non-anaemic patients is even higher at 57% in men and 79% in women [18]. Iron status, independently of haemoglobin (Hb) levels, is associated with reduced exercise capacity, impaired quality of life and increased risk of death and hospital (re-)admissions [12,14,16,19,20,21]. Intravenously correcting ID was shown to improve quality of life, exercise performance and symptoms regardless of Hb levels [22,23,24,25]. These observations underscore the crucial role of iron beyond erythropoiesis [26]. Additionally, although the recent AFFIRM-AHF study statistically missed its primary endpoint (i.e., combined recurrent hospitalisations or cardiovascular mortality), administering intravenous (IV) iron in patients with acute HF significantly reduced recurrent hospitalisations [27,28]. The evidence on mortality is yet to be ascertained [29].

The mechanisms leading to these clinical benefits, as well as the underlying causes that led to ID in the first place, remain poorly characterised. Not understanding the pathomechanisms leading to ID might potentially result in missed diagnoses (i.e., underlying gastro-intestinal malignancies, malnutrition) and undertreatment [30,31]. Recently, ID was identified as a key element involved in the pathophysiology of HF and its progression, suggesting that ID might be more than merely a comorbidity in HF [32,33,34]. In this review, we aim to summarise the current understanding of the pathophysiology of ID as well as its biological repercussions in patients with HF. Moreover, the latest progress in mechanistic studies on the potential role of IV iron supplementation is outlined.

### 1.1. Physiologic Roles and Regulation of Iron

**Figure 1:**
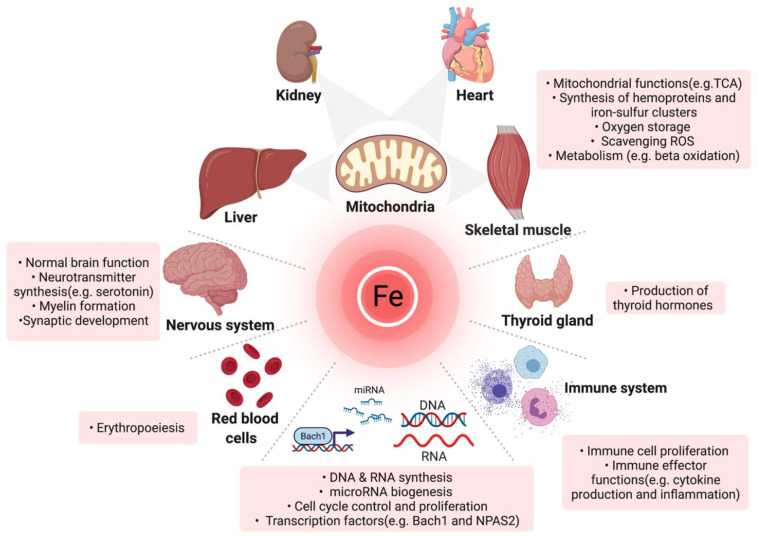
Overview of the multifaceted roles of iron in diverse organs and molecular processes. TCA: tricarboxylic acid cycle; miRNA: microRNA, ROS: reactive oxygen species (Created with BioRender.com, accessed on 24 November 2021).

Iron is an essential cofactor for the normal functioning of many enzymes participating in vital cellular and organismal functions, making it indispensable for every living cell [35,36,37]. In addition to its important role in oxygen transport and storage as a constituent of hemoglobin and myoglobin respectively, iron is crucial for many enzymes and proteins involved in oxidative metabolic processes (e.g., mitochondrial respiratory chain, oxidative enzymes and protection against oxidative stress), microRNA biogenesis, the function of the thyroid gland, central nervous system and immune system [35,36,38]. Furthermore, iron is crucial for the synthesis and degradation of proteins, lipids (e.g., β-oxidation of fatty acids), carbohydrates, DNA and RNA [38,39,40,41,42]. Of note, iron is particularly important for cells either with high energy demand (cardiomyocytes, hepatocytes, neurons, renal and skeletal cells) or high mitogenic activity (e.g., haematopoietic and immune cells) [26,43]. Accordingly, these cells are more sensitive to ID [26]. An overview of the functions and proteins that require iron are outlined in Figure 1 and Table 1 [43,44,45], respectively.

Besides its crucial roles in the body, iron is a potentially harmful element to cells given its chemical reactivity and its propensity to generate reactive oxygen species through participating in Fenton’s reaction [35,43]. Iron is almost always linked to either ferritin intracellularly or extracellularly to transferrin as free iron ions are toxic [35]. In addition, iron status is tightly regulated both systematically by hepcidin and cellularly through iron-regulatory proteins. A detailed description of iron metabolism is beyond the scope of this review. A comprehensive overview of cellular and systemic iron metabolism can be found elsewhere [45,46,47,48].

### 1.2. Definition of Iron Deficiency

ID ensues when iron supply is insufficient to meet the body’s needs or to cover the iron lost physiologically or pathologically [39]. ID may manifest itself in two distinct forms with intertwined pathophysiology, namely functional and absolute ID. Absolute ID (AID) reflects depleted iron stores, while functional ID (FID) is characterised by reduced availability of iron despite sufficient or overly abundant iron stores due to suboptimal iron trafficking induced by hepcidin. It is crucial to note that studies in HF differ in defining ID [10,49,50,51]. The most widely used definition of ID, which is also adopted by the European Society of Cardiology (ESC), is a ferritin level <100 μg/L (reflecting AID) or ferritin (100 to 300 μg/L) with a transferrin saturation (TSAT) <20% (reflecting FID) [8]. This definition of ID has, however, been criticised as it has never been validated against a gold standard and remains a subject of considerable debate, especially in patients with acute HF [49,50,52,53,54,55].

The ESC definition of ID (also called the FAIR-HF definition [22]) is limited by relying heavily on ferritin levels, thereby labelling patients with TSAT ≤20%, but a ferritin >300 μg/mL as iron sufficient, while labelling those with isolated hypoferritinaemia (ferritin <100 μg/mL with a TSAT >20%) as iron deficient. This latter category was found to be iron sufficient when compared to bone marrow staining, the golden standard for diagnosing ID [54]. Although ferritin is one of the most widely used biomarkers to detect iron deficiency [56], serum ferritin levels can be profoundly influenced by several factors such as inflammation, infection and malignancy, making it falsely elevated in an inflammatory state such as HF and thus does not correlate with iron availability [57]. We showed that, compared to bone marrow iron staining, the ESC definition of ID has a sensitivity of 82.4% and a specificity of 72% for detecting ID in patients with HF [54]. Serum iron (≤13 μmol/L) and TSAT (≤19.8%) were significantly better cutoffs than the FAIR-HF definition, with areas under the curves (AUC) of 0.922 and 0.932, respectively. Adding ferritin to either definition did not result in a significant increase in the AUC, suggesting that ferritin does not contribute to more accurate identification of truly iron deficient HF patients, and as such, both serum iron ≤13 μmol/L and TSAT ≤19.8% are good indicators of ID as standalone. Prognostically, these two definitions are independently associated with a higher incidence of all-cause mortality, while isolated hypoferritinemia did not [54]. Several studies corroborated these findings [10,49,51,54,58,59,60]. More recently, it was found that persistent ID (defined as a serum iron ≤13 µmol/L) was associated with poor prognosis, while its resolution was associated with improved outcomes. Similar trends were found when defining ID as TSAT <20%, but not when defined as per the FAIR-HF criteria [49]. Remarkably, Cleland et al. found that higher ferritin levels (and not lower levels) were significantly associated with a higher risk of all-cause or cardiovascular mortality, further questioning the correlation between ferritin and iron availability in patients with HF. Additionally, subgroup analysis of individual patient data meta-analysis (*n* = 839) pooled from four double-blind, randomised controlled trials (RCTs) showed that although intravenous ferric carboxymaltose (FCM) generally reduces recurrent cardiovascular hospitalisations and cardiovascular mortality, patients with TSAT <20.1% benefit more from FCM iron than those with TSAT >20.1% even if ferritin levels were low [61]. Similarly, in the IRON-CRT trial, it was found that HF patients with TSAT <20% benefit more from FCM iron than if TSAT was >20% in terms of cardiac contractility and left ventricular ejection fraction (LVEF) [62]. However, similar interaction was not found in the AFFIRM-AHF trial [27]. The aforementioned findings confirm the accuracy of TSAT <20% and serum iron ≤13 µmol/L in identifying truly iron deficient HF patients while questioning the diagnostic and prognostic usage of ferritin in detecting ID in HF.

On the other hand, these two definitions of ID might have their own limitations, as serum iron is subjected to circadian variations [63], and TSAT might be falsely elevated in malnutrition and advanced stages of renal insufficiency [50,64,65,66]. Recent studies proposed serum soluble transferrin receptor (sTfR) as the most auspicious novel ID-related biomarker since circulating sTfR levels reflect the iron demand of the body in addition to the erythroid proliferation rate quantitatively [67]. In a similar approach to our bone marrow study, Sierpinski et al. found that ID defined as serum sTfR of ≥1.25 mg/L is more accurate in identifying ID when compared to bone marrow staining in clinically stable patients with HF [55]. Of note, adding sTfR to multivariable models for predicting 3-year all-cause mortality in patients with HF abolishes the prognostic value of serum ferritin and TSAT after adjusting for all other prognosticators. These findings suggest that elevated serum sTfR is a better surrogate for depleted intracellular iron. In line with these findings, Leszek et al. found that only serum sTfR significantly correlated to myocardial and mitochondrial iron status, but not ferritin, serum iron or TSAT [68], indicating that sTfR reflects tissue iron demands more accurately. Nevertheless, the lack of assay standardisation restricts its implementation in clinical routines [66].

To summarise, defining ID in HF using classical biochemical iron parameters appears to be not straightforward. Mounting evidence suggests that ferritin should not be taken into consideration when diagnosing ID in patients with HF but may be used as a safety parameter to avoid the administration of iron to patients with potential iron overload; TSAT or serum iron alone are better indicators of systemic ID, while sTfR might outperform them all. Although the current ESC definition of ID performed thus far reasonably good in general, it is broad and unspecific in identifying those who are truly iron deficient; the high prevalence of ID in HF might have precluded the importance of choosing a more accurate definition to identify those who are truly iron deficient and need IV iron. Identifying truly iron-deficient patients is crucial as inaccurate diagnoses of ID [49] and unnecessary treatment with FCM might dilute the benefits of IV iron and lead to increased risks such as hypophosphatemia [69]. Furthermore, in light of existing evidence indicating different mechanisms leading to myocardial and systemic ID [70] (as discussed below) as well as poor accuracy of systemic biomarkers in detecting myocardial ID, which might be a major driver behind clinical improvements upon iron supplementation, future studies should evaluate other ID-related surrogates in order to identify HF patients that might benefit from iron supplementation on a systemic and cellular level.

## 2. Causes of Iron Deficiency in Heart Failure

Replenishing iron stores using IV iron addresses the consequence of ID, but not the cause. Accordingly, the underlying aetiology should be investigated, as also indicated in the guidelines [8]. However, from these guidelines, it is unclear which causes should be sought for and which examinations should be performed.

The underlying causes of ID are poorly characterised. Multiple mechanisms are likely to be operational. A variety of postulated mechanisms have gained great attention to explain the high prevalence of ID in HF beyond anaemia. Several factors were shown to be independently associated with ID in HF, including advanced age, kidney failure, female gender, malnutrition, chronic inflammation, reduced iron absorption, increased iron loss and heart failure severity [15,16,26,51]. Of note, many of the aforementioned risk factors are postulated based on observational studies and have not yet been confirmed as a cause in patients with HF and thus, remain hypothetical.

Since iron status is the resultant of inflow and outflow, the causes of iron deficiency in HF can be classified into three categories: (1) reduced iron intake, (2) reduced iron absorption and (3) increased iron loss. In addition, specific causes of myocardial iron deficiency are discussed in the following sections.

### 2.1. Reduced Iron Intake and Low Iron Bioavailability

In a large international cohort of 2357 patients with worsening HF, decreased estimated protein intake was used as a surrogate to estimate dietary iron intake. It was found that patients with lower estimated protein intake have a higher prevalence of ID (particularly absolute ID [71]), indicating poor nutritional status as a potential cause of ID in HF [51]. Studies show that approximately 35% to 78% of HF patients suffer from malnutrition, which has also been independently associated with adverse outcomes [72,73,74,75]. The aetiology of malnutrition is complex and thought to be multifactorial where fatigue, dyspnoea, swallowing disturbances, nausea, anxiety, food monotony, reduced appetite and early satiety could play a role [76,77,78]. The advice to reduce dietary salt intake (albeit controversial [79,80]) could compromise the nutritional status of individual patients with HF [81,82]. Dietary intake of iron in HF patients assessed using a 4-day food diary is lower than the recommended intake in 46% of patients, with significantly lower intake in patients with NYHA class III–IV as compared to class II [83]. This suggests that malnutrition and subsequently lower iron intake is more severe as HF progresses. Other studies have also found inadequate iron intake in patients with HF, especially among women above 50 years [84,85].

Iron absorption is closely tied to iron bioavailability, which is defined as the extent to which iron is absorbed from the ingested diet. Iron bioavailability is dependent on the form of iron consumed, haeme or non-haeme iron, which have different mechanisms of absorption [86]. Haeme iron, which is found in haemoglobin and myoglobin of animal food sources, is readily absorbed and contributes to 20–30% of the total absorbed iron [87]. Unlike haeme iron, intestinal absorption of non-haeme (derived from plants and iron-fortified foods) is less readily absorbed and is dependent on the balance between absorption inhibitors and enhancing factors [86]. Ultimately, diets low in iron bioavailability, even if they consist of sufficient absolute amounts of iron, may lead to ID [88].

With the exception of calcium, which inhibits both forms of dietary iron, phytates and polyphenols (found in tea, coffee or chocolate) inhibit the absorption of non-haeme iron [86]. Vitamin C and consumption of meat enhance absorption of non-haeme iron even in a plant-based phytate-rich diet [89]. Diet has been implicated as a risk factor for developing ID in HF. Multiple studies showed a higher prevalence of ID in Indians compared to other Southeast Asian and European populations [17,20,90,91,92], which potentially can be explained by dietary habits since vegetarianism and drinking tea is very common among the Indian population [93]. Besides containing mainly non-haeme iron, a plant-based diet is full of phytates and polyphenols, which are known potent inhibitors of non-haeme iron absorption, making vegetarians more prone to develop ID [87,94,95]. In a study of 226 HFrEF patients from the Himachal Pradesh heart failure registry, it was found that HF patients that consume a vegetarian diet were 2.5 times more likely to have ID (odds ratio 2.5 (95% confidence interval 1.4–4.6)) [96]. Therefore, poor diet and dietary habits seem one of the risk factors predisposing to ID in HF, and accordingly, recommending adequate nutritional intake might be crucial non-pharmacological advice for tackling ID in HF.

An alternate explanation of the inter-ethnic differences in ID prevalence could be the genetic makeup [39,97]. Individuals with mutations in the TMPRSS6 gene have higher hepcidin levels, preventing normal absorption of dietary iron and leading to iron-refractory IDA [39]. A meta-analysis of genome-wide association studies has shown that common variants in TMPRSS6 (especially the rs855791 SNP) were associated with lower Hb and ferritin but higher serum transferrin receptor and transferrin concentration, indicating susceptibility to ID. The minor allele frequency (MAF) of rs855791 varies across ethnic groups significantly, with the highest MAF being in Asian populations (0.55 in Asians vs. 0.42 in Caucasians, *p* ≤ 0.0001) [98]. Other variants were also found to have ethnic differences [97]. These findings, together with the dietary habits, could predispose some populations more to ID. No study has yet investigated the relationship between the genetic makeup of HF patients in relation to ID.

Increasing Iron Intake: Is It Effective?

Increasing iron intake orally in HF patients using Forceval (mix of micronutrients including 12 mg of iron) for 12 months did not significantly increase serum ferritin or serum iron [94]. Furthermore, in the IRONOUT-HF study, it was found that taking oral iron polysaccharide tablets (150 mg twice daily) for 16 weeks did not improve functional capacity and quality of life in patients with HFrEF in spite of the minimal improvement in iron stores [95]. It is worth noting that the cumulative amount of oral iron received within the study period exceeds the recommended dosage intravenously to correct ID by more than 15 times [8,95]. Knowing that the main source of iron used by the body is endogenously obtained as a result of scavenging iron from senescent erythrocytes by macrophages [67], these results may implicate mechanisms other than insufficient dietary iron intake alone as a cause of ID in HF.

However, oral iron preparations differ in their tolerability, bioavailability and efficacy in correcting ID as they differ in their routes of absorption [99,100]. A recent small (*n* = 50) non-randomised open-label study showed that 3-month therapy using another formulation of oral iron (Sucrosomial iron) in iron-deficient HFrEF patients was significantly associated with improved iron indices, exercise capacity and quality of life [101]. Whether oral Sucrosomial iron (SI) can be used as a stand-alone or adjuvant therapy to IV iron supplementation is currently unknown. Two ongoing randomised controlled trials in HFpEF (PREFER-HF, NCT03833336) and HFrEF (IVOFER-HF, EudraCT 2017-005053-37) patients comparing FCM with SI iron will shed more light on the efficacy of SI.

### 2.2. Reduced Iron Absorption

In healthy individuals, iron absorption is determined by the form of iron consumed, iron absorption enhancers and/or inhibitors and iron status [45]. The physiological response to ID in otherwise healthy individuals is increased absorption of dietary iron [39]. Although HF patients are usually malnourished and the iron intake is suboptimal [73,84,86], the cause of ID in these patients is probably not solely due to decreased intake. Reduced iron absorption is thought to be an important factor in causing ID in HF as it can explain why IV iron works in replenishing iron stores in iron-deficient HF while particular oral iron preparations do not [102]. IV iron bypasses regulatory mechanisms that are thought to reduce iron absorption in HF, overcoming the absorptive inflammatory blockade of intestinal iron absorption [103]. Several factors are thought to reduce iron absorption, leading to ID in HF.

#### 2.2.1. Impaired Intestinal Function

ID is associated with right ventricular dysfunction [104], peripheral oedema and orthopnoea in HF [10,51], possibly implicating congestion in the pathogenesis of ID in HF [105]. HF patients have an altered intestinal morphology, permeability and absorption [106]. HF patients with venous congestion have reduced blood to the intestine, causing intestinal hypoperfusion and consequently nonocclusive bowel ischemia, increased mucosal permeability, bowel edema, cachexia and altered composition of mucosal bacteria [106,107,108,109,110]. Collectively, all of this might culminate in the malabsorption of micronutrients, including iron [111].

A study showed that in rats with IDA, morphological and functional compensatory mechanisms are important adaptive mechanisms to increase intestinal absorption of iron [112,113]. These mechanisms include increased cell proliferation, mucosal thickness, epithelial surface area, villus length and width. Given the altered intestinal morphology and function in HF, these adaptive mechanisms might not be operational to correct ID physiologically [114].

In addition to impaired intestinal morphological adaptations in patients with HF, studies in animals showed that adaptive transcriptional mechanisms to counteract ID are defective in HF models. Unlike IDA rats without HF, in Dahl salt-sensitive HF rats, it was found that intestinal expression of important genes for intestinal iron absorption such as duodenal cytochrome b (Dcyt-b), divalent metal transporter 1 (DMT-1) and ferroprotein was not upregulated in spite of reduced hepcidin expression [114]. Remarkably, the expression of intestinal hypoxia-inducible transcription -2 (HIF-2α) did not increase in IDA-HF rats but increased in IDA rats without HF [114]. Upregulation in intestinal HIF-2α is an essential adaptive mechanism to counteract ID by increasing iron absorption [115,116]. Taken together, this suggests an abnormal iron regulating system in HF, which impairs adaptive responses to correct ID physiologically. Interestingly, gut microbiome-derived metabolites are capable of modulating HIF-2α signaling [117,118]. There is accumulating evidence implicating changes in the composition of the gut microbiome in the pathogenesis and perpetuation of HF [119]. Whether gut dysbiosis observed in patients with HF plays a role in ID pathogenesis is currently unknown.

A recent study, however, albeit small sample size (*n* = 30), showed that patients with HF and ID have higher oral iron absorption compared with age and sex-matched control subjects without ID [120]. Nonetheless, it is not clear whether this increase in absorption is due to HF or ID itself, as there was no comparison between subjects with ID and no HF to indicate whether HF might impair oral iron absorption. Larger studies are needed to ascertain the causal relationship between impaired absorption of iron and the development of ID in HF.

#### 2.2.2. Inflammation: Role of Interleukin-6 and Hepcidin

HF is associated with an inflammatory state that results in increased production of inflammatory markers like interleukin-1(IL-1), interleukin-6(IL-6) and tumour necrosis factor alpha (TNF-α). Inflammation is thought to play a role in causing ID in several chronic inflammatory diseases (such as cancer [121], CKD [122,123] and rheumatoid arthritis [124]), including HF, with hepcidin having a pivotal role herein [15,20,26,51,53,125,126,127]. IL-6 is the main inflammatory trigger of increased hepcidin expression [128]. Other cytokines can also induce hepatic hepcidin expression indirectly by upregulating IL-6 such as TNF-α, interferon alpha, activin B [129], or directly independent of IL-6 such as oncostatin M [130] and interleukin-22 [131]. Hepcidin internalises and degrades the only known iron exporter, ferroportin. Thereby, increased hepcidin levels lead to a decrease in iron absorption and mobilisation from the reticuloendothelial system, causing predominantly functional ID in HF [35,129].

Increased hepcidin levels have been directly linked to the pathogenesis of IDA associated with chronic inflammatory diseases such as cancer [121], CKD and autoimmune diseases [124,132]. In HF, however, data on associations between hepcidin, inflammation and ID are not consistent. In acutely decompensated HF patients admitted to the hospital, the prevalence of ID decreased after 30 days of discharge. This change in iron parameters was correlated, albeit weakly, with changes in inflammatory markers suggesting that inflammation may influence iron status [133]. In line with these findings, increased plasma IL-6 concentration was found to be associated with lower iron levels and higher hepcidin levels in an international cohort containing 2329 HF patients [134]. In contrast, a study by Jankowska et al. found an inverse relationship between IL-6 and hepcidin [132]. However, in the BIOSTAT-CHF cohort, iron-deficient HF patients (defined as TSAT < 20) were found to have lower hepcidin levels with higher levels of inflammatory markers (CRP) compared to patients without ID [51]. Subsequent analysis showed that higher hepcidin levels are significantly associated with a higher prevalence of FID but a lower prevalence of AID [71]. Many studies found no independent association between ID and inflammation when no distinction was made between FID and AID [16,132]. This may suggest differential involvement of inflammation in the pathophysiology of the two types of ID.

Reduced hepcidin levels in IDA have already been documented in animals [114] and human studies [70,132,135,136,137,138]. In patients with stable HF, an inverse relationship between hepcidin levels and HF severity was found, with levels of hepcidin in patients with NYHA class IV being significantly lower even when compared to healthy controls [132]. Whereas progression of HF is associated with heightened inflammatory response and increased prevalence of ID [15,16], hepcidin levels seem to decrease despite increased proinflammatory cytokines [132].

Increased inflammation, disordered iron balance, yet low hepcidin. What could explain these contradictory results? First, ID seems to have a more profound impact on hepcidin expression levels than inflammation as hepcidin expression is regulated by multiple pathways; depleted iron stores, hypoxia and ineffective erythropoiesis all decrease the expression of hepcidin, while inflammation induces its expression [139]. Although hepatic hepcidin expression is influenced by multiple factors, low plasma levels of hepcidin specifically reflect depleted iron stores [48,50]. The strength of each stimulus determines the expression of hepcidin rather than by an absolute hierarchy among signaling pathways [140]. Another possible explanation for the observed low hepcidin levels might be the chronological aspect of ID development. In other words, ID in HF may start being functional since high hepcidin levels were found in the early stages of HF [132], and if it lasts for a long time, absolute ID ensues. Future studies should investigate the sequence of changes in iron status in relation to the two types of ID in HF patients.

#### 2.2.3. b: Role of TNF-α

Lastly, other inflammatory markers may also lead to impaired iron metabolism in HF independently of hepcidin. In vitro models showed that treating immortalised human gastrointestinal epithelial cells with TNF-α led to reduced iron uptake significantly [141]. In mice, intraperitoneal injection with TNF-α caused ID by reducing intestinal iron absorption through decreasing the expression of DMT1 with no changes in hepatic hepcidin production [142]. However, Weber et al. showed in a relatively small cohort of HF patients (*n* = 60) that TNF-α was not significantly higher in HF patients with ID [135], which may suggest that TNF- α might not be the main driver behind ID in HF. However, convincing data on the relevance of this mechanism in leading to ID in patients with HF is lacking.

#### 2.2.4. Hypochlorhydria and Excess Alkalinisation

Gastric acids play an important role in absorbing non-haeme iron [143]. A low pH is essential to convert non-bioavailable ferric (Fe^3+^) iron to ferrous (Fe^2+^) iron by ascorbic acid or Dcytb prior to absorption by DMT1 [144]. Impaired gastric acid production as a result of chronic use of gastric acid-inhibiting medication such as proton-pump inhibitors (PPIs) and histamine-2 receptor antagonists(H2Ras) reduces oral iron absorption greatly. PPIs and H2Ras increase pH and thereby reduce iron absorption [39]. These medications are routinely prescribed in clinical practice for treating peptic ulcers, dyspepsia, gastroesophageal reflux disease and/or prevention of gastrointestinal bleeding in patients using antiplatelets such as clopidogrel and/or anticoagulants such as warfarin. In the general population, chronic use of antacids was associated with an increased risk of developing ID [145,146]. Remarkably, using PPIs significantly inhibits the absorption of non-haeme iron even in patients with hereditary hemochromatosis where iron absorption is increased [147]. In a study comprised of >22,000 patients with HF, it was found that the use of PPIs is common (29%), and the prevalence of anaemia among PPIs users was significantly higher [148]. However, only one study showed that iron-deficient HF patients were more likely to also use PPIs [51]. This association lost its significance after adjustments in multivariable logistic regression. PPIs are often co-prescribed with medications that cause GI bleeding, such as antiplatelets and/or anticoagulants [148], as well as NSAIDs (potential confounders) [51]. Hence, coexisting blood loss cannot be excluded as a cause of ID in HF, as explained below.

### 2.3. Increased Iron Loss

Gastrointestinal Blood Loss

Observational studies show that the prevalence of ID is substantially higher in older HF patients [15,16,51,149]. Most patients with HF are elderly [4]. ID in the elderly is almost always due to non-dietary factors [150,151], with colorectal cancer, colonic polyps and angiodysplasia being the most common causes [39]. Other potential causes of occult upper and lower GI bleeding include esophagitis, gastritis, peptic ulcer, or inflammatory bowel disease [152,153]. In developing countries, hookworm infection could also lead to iron loss [39]. In otherwise healthy individuals presenting with idiopathic IDA with no GI symptoms, more than half of the patients have GI abnormalities upon endoscopic investigations [149,154,155]. Another study, including 151 individuals with ID older than 70 years (83 ± 6 years), found a higher prevalence of GI lesions irrespective of anaemia (66% in IDA vs. 65% in iron-deficient nonanemic patients) [156]. Of note, GI malignancies (gastric, oesophageal or colon cancer) were also found in 15% of them, independently of the presence of anaemia. Similar findings were also found in a larger Korean cohort [157]. In a study conducted by Martens et al. including 699 HF patients who underwent a full endoscopic workup, it was found that GI malignancies in iron-deficient HF patients were present in around 10%, with no statistically significant difference between those with IDA and ID without anaemia (9.3% vs. 10.5%, respectively; *p* = 0.551) [158].

Occult blood loss can be aggravated using medications. HF patients are more vulnerable to GI lesions compared to the general population as they have multiple risk factors for GI bleeding, including older age, multimorbidity and polypharmacy of medications that cause intestinal abnormalities. Antiplatelets (e.g., aspirin [159] and clopidogrel [160]) and/or anticoagulants (e.g., warfarin [161]) are known to increase GI bleeding by causing GI lesions, especially if used in combination [162,163,164]. Such complex dual and triple antithrombotic drug combinations increase the risk of GI bleeding substantially [165,166]. Remarkably, the presence of comorbidities amplifies the risk of GI bleeding when these agents are used, with HF being an independent risk factor [162,167,168,169]. This might suggest that HF patients have a vulnerable gastric mucosa, and using such medications to treat another comorbidity in HF makes them more susceptible to GI lesions. However, associations between ID in HF and the use of antiplatelet and/or anticoagulants are not consistent. Some studies found no association between ID and the use of these medications [15,16,20], while only one study identified P2Y12 inhibitors as an independent predictor of ID [51].

Contrastingly, increasing evidence suggests that patients with HF have an increased risk of developing cancer [163,164,170]. Interestingly, iron has emerged as a potential cause for tumorigenesis, especially in colorectal cancer [171,172,173,174]. The contribution of ID to the incidence of cancer in HF patients is not yet elucidated and worth further investigation.

Altogether, elderly HF patients are at an increased risk of chronic GI bleeding, which is very likely to result in ID. In these patients, identification of ID should prompt further investigations as ID is most of the time indicative of underlying pathology, which might be potentially reversible.

### 2.4. Systemic vs. Myocardial Iron Deficiency: Role of Neurohormonal Activation

The aforementioned causes considered ID as extra-cardiac comorbidity that has an impact on patients with HF. However, mounting evidence indicates differences in mechanisms leading to cellular and systemic ID [175,176,177]. In HF, it is was found that myocardial ID (MID) is poorly related to systemic iron homoeostasis biomarkers [68,70], suggesting that MID may be caused by other mechanisms other than reduced systemic iron availability [178,179]. Petrak et al. revealed that hearts of volume overload-induced HF rats develop MID despite normal iron absorption and preserved systemic iron levels [180].

Neurohormonal activation, which is a hallmark feature of HF [181], is thought to be the main cause behind myocardial ID. Maeder et al. showed lower iron content in the myocardium of patients with HF correlating with a lower mRNA expression of transferrin receptor 1 (*TFR1*), which is the main pathway by which iron enters cardiomyocytes [182]. Experimentally, using cardiomyocytes from neonatal rats, they found that a reduction in TFR1 expression is linked to increased activation of the neuroendocrine system, particularly aldosterone and norepinephrine [182]. This suggests that myocardial ID is likely to be caused via neurohormonal activation by downregulating TFR1. Furthermore, HF patients with MID had a lower rate of using beta-blockers compared to non-MID HF patients [183]. Moreover, Haddad et al. highlighted the importance of iron-regulatory proteins (IRP-1 and IRP-2) to the heart in securing myocardial iron [178]. More recently, Tajes et al. demonstrated in mice with isoproterenol (β-adrenoceptor agonist)-induced HF that neurohormonal activation leads to MID accompanied by mitochondrial dysfunction by reducing extracellular iron uptake and increasing intracellular iron release [33]. These findings were validated using embryonic rat heart-derived H9c2 cells challenged with norepinephrine and/or angiotensin 2, which led to similar results [33]. This implies that neurohormonal activation worsens HF by decreasing myocardial iron, suggesting that ID may be more than merely a comorbidity. In line with these findings, cardiac iron content is two times lower in patients with end-stage HF compared to non-advanced HF (New York Heart Association class II or III) with reduced ejection fraction, which may suggest that MID develops or worsens during the course of HF [70].

These pre-clinical findings raise an intriguing hypothesis that systemic iron replacement may benefit patients with HF before the onset of systemic iron deficiency. The proactive approach of IV iron in patients with HF without apparent systemic IV remains to be investigated. In chronic kidney disease (CKD), the PIVOTAL trial demonstrated that in patients undergoing maintenance haemodialysis, high dose proactive IV iron sucrose (for those with ferritin <700 ng/mL and TSAT <40%) showed both noninferiority and superiority for the primary endpoint (composite of death and major adverse cardiovascular events) compared to reactive low dose IV iron (for those with ferritin <200 ng/mL or TSAT was <20%) with no significant difference in infection rate [184]. In addition, this proactive approach resulted in significantly fewer hospitalisations for HF, lower doses of erythropoiesis-stimulating agents used and a lower incidence of blood transfusion. Although this trial was not in HF patients, it does show benefits even in those seemingly iron-sufficient patients based on traditional definitions.

**Figure 2:**
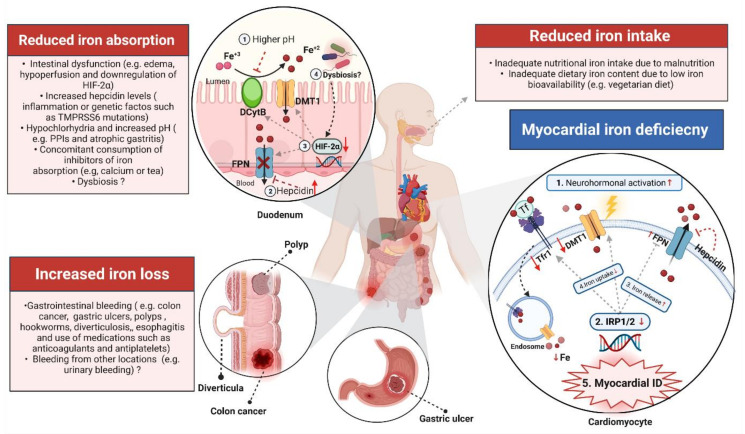
Summary of the current understanding of the mechanisms underlying iron deficiency in heart failure. This step is essential to enable iron uptake by the divalent metal transporter (DMT1). This reduction process is influenced by the pH of the luminal contents, and thus, any factors that influence the pH, such as proton pump inhibitors, can impair non-haeme iron absorption. Within the enterocyte, iron can be stored in ferritin or exported into the bloodstream by the iron exporter ferroportin (FPN), which is controlled by hepcidin. Increased hepcidin levels internalise FPN, leading to sequestration of iron within the enterocytes and thus impairing absorption of iron to the blood. HIF-2 regulates transcription of DCytB, DMT1 and FPN iron transport machinery. Downregulation of HIF-2 can lead to a dysfunctional iron regulating system in HF. Gut microbial metabolites can decrease HIF-2 expression and thus may influence systemic iron homeostasis. The third mechanism that could also result in systemic ID is increased iron loss due to gastrointestinal pathology such as colon cancer. In the heart, neurohormonal activation leads to myocardial ID by downregulating iron-regulatory proteins (IRP1/2). Iron circulation in the heart is controlled by IRP1/2 and hepcidin. In turn, this defective downregulation of IRP1/2 as well as hepcidin leads to increased iron release (as a result of decreased hepcidin levels and thus higher FPN) and decreased iron uptake due to downregulation of transferrin receptor 1 and DMT1. (Created with BioRender.com, accessed on 24 November 2021).

It should be emphasised that it is yet to be determined whether deregulation in intrinsic myocardial iron metabolism could lead to systemic ID in patients with HF. A cross-sectional study composed of 742 HF patients found an independent association between increased sympathetic activation and systemic ID as measured by the increased plasma norepinephrine levels [32]. More confirmatory studies are needed to answer whether MID caused by neurohormonal activation is an independent abnormality or a contributing factor towards systemic ID in patients with HF. An overview of the discussed mechanisms leading to ID in HF is summarised in Figure 2.

## 3. Deleterious Biological Consequences of Iron Deficiency

**Figure 3:**
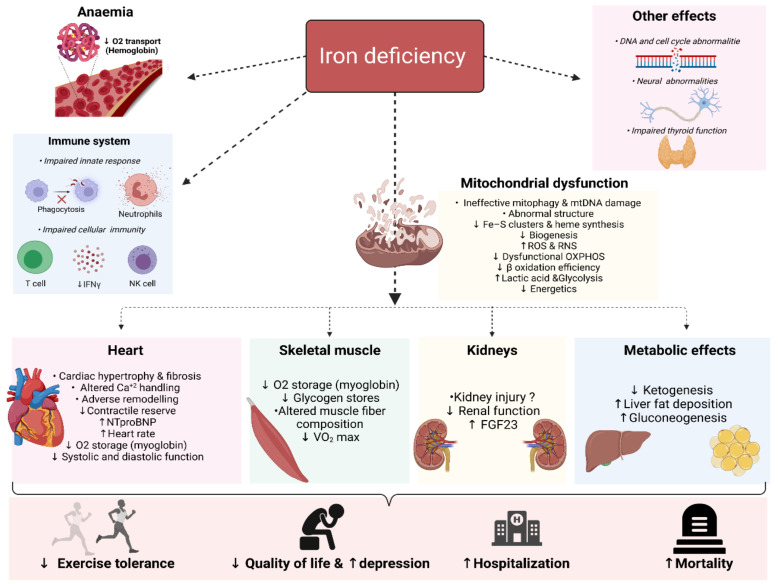
Overview of the biological consequences of iron deficiency. NTproBNP: N-terminal pro-b-type natriuretic peptide; OXPHOS: Oxidative phosphorylation; RNS: reactive nitrogen species; ROS: reactive oxygen species and FGF23: Fibroblast growth factor-23 (Created with BioRender.com, accessed on 24 November 2021).

The functional and clinical impairments as well as the benefits of correcting ID observed in non-anaemic iron-deficient HF patients point towards the important role of iron in nonhematopoietic tissues [16,20,21,23,185,186]. The underlying mechanisms between the worst symptomatic status and prognosis remain poorly characterised. Recent years sparked an avalanche of research seeking to explain the mechanisms by which ID contributes to these adverse effects. In order to understand the consequences of ID in HF patients beyond anaemia, consideration should be directed towards the roles of iron beyond mere erythropoiesis. In the following sections, the cellular and subcellular effects of ID are discussed. A summary of the deleterious effects of ID is shown in Figure 3.

### 3.1. Mitochondrial Function and Metabolic Effects

Cells with high energy demand such as cardiomyocytes, hepatocytes, nephrons and skeletal myocytes are abundant with mitochondria. Mitochondria are the major intracellular sites of iron utilisation and accumulation as they are the sites where synthesis of haeme and iron–sulphur clusters takes place [187,188]. Beyond its biosynthetic role in haeme and iron–sulphur clusters, iron was also shown to be crucial for mitochondrial biogenesis as ID affects both iron-containing and non-iron-containing mitochondrial proteins, indicating a reciprocal relationship between adequate iron content and mitochondrial function [189].

Mitochondria are the primary combustion machinery in cells for burning fuel such as glucose, fatty acids and ketone bodies. The final step to producing adenosine triphosphate (ATP) from these nutrients is oxidative phosphorylation (OXPHOS), for which sufficient iron (in addition to other pathways) is vital. This fact highlights how crucial iron is for proper energetics in all cells.

Besides producing ATP, mitochondria are also involved in controlling cellular Ca^2+^ [190], generating reactive oxygen species, cellular death, synthesis of pyrimidine, amino acids and lipids [187]. Accordingly, deficiency in iron impairs mitochondrial function at many levels. ID has been linked to morphological changes in the mitochondria, such as an increase in size and a decrease in cristae [191], as well as functional changes such as reduced production of ATP [192], mitochondrial DNA damage [191], increased gluconeogenesis [187,193], increased lactic acid production [185,186,194], reduced mitochondrial biogenesis and impaired mitophagy [179,189], increased mitochondrial cytochrome c release (and hence apoptosis) and reactive nitrogen species expression [195,196,197,198,199,200]. All of this culminates in mitochondrial damage. As such, ID may worsen HF by causing mitochondrial damage [201,202], and its correction augments mitochondrial function.

Oxidative Stress

Mitochondria are the primary source of production and scavenging of both reactive oxygen species (ROS) and reactive nitrogen species (RNS) [203,204]. Not only can iron excess lead to oxidative stress via Fenton-type reactions but ID was also shown to promote oxidative and nitrosative stress [204]. This is thought to be related to the reduced antioxidant activity (e.g., catalase enzyme) [205] and increased superoxide production as a result of mitochondrial dysfunction. In the heart samples of HF patients undergoing transplantation, myocardial ID is associated with reduced expression of key protective enzymes that scavenge ROS, such as catalase, glutathione peroxidase and superoxide dismutase 2 [183]. This may point towards ROS and/or RNS induction as an adverse consequence of ID, which is seen as one of the underlying mechanisms leading to myocardial remodeling and HF progression [206]. Toblli et al. showed that even without completely correcting anaemia, IV iron sucrose reversed the anaemia-induced cardiac remodeling, prevented cardiac fibrosis and improved cardiac function by mitigating oxidative/nitrosative stress and inflammation in the heart [207]. Furthermore, in another rat model of HF, intravenously administering iron resulted in higher tissue activity of the antioxidant superoxide dismutase [208]. A recent study found similar results in a mice model of myocardial infarction [209]. Several studies showed that ID participates in the induction of oxidative stress in many organs, including the liver and the kidneys [210,211]. All in all, ID makes cells more prone to oxidative/nitrosative damage, and its correction may ameliorate these adverse effects of ID.

### 3.2. Heart

Since the heart has the highest energy expenditure of all organs [212], intact performance of cardiomyocytes is inextricably linked to mitochondrial function, for which sufficient iron is vital. This makes cardiomyocytes especially susceptible to the adverse effects of ID. Depriving human cardiomyocytes of iron leads to impaired contractile function with reduced activity of respiratory complexes I, II and III [192]. Remarkably, these adverse effects of ID can be reversed by iron supplementation. Similarly, several animal studies showed that systemic ID, even without anaemia [213], is associated with structural changes in the heart, including cardiac hypertrophy, irregular sarcomere organisation, mitochondrial swelling, left ventricular(LV) dilation , LV hypertrophy, lung congestion and cardiac fibrosis [195,214,215,216]. In addition to structural remodelling, the hearts of mice with IDA exhibit a hypoxic phenotype and altered Ca^2+^ handling, with a metabolic shift towards lactic acid-producing glycolytic metabolism [185,217]. Furthermore, hearts of mice models with isolated myocardial ID without anaemia develop cardiomegaly, impaired contractile function, shifts towards anaerobic respiration, dysfunctional oxidative phosphorylation and impaired mitophagy despite normal systemic iron levels [178,179].

Failing human hearts are characterised by reduced *total* iron content [69,193,218]. Systemic ID and/or MID is associated with worse cardiac function, diminished contractile reserve [219], decreased mitochondrial enzymatic activities of both oxidative phosphorylation and anti-oxidative enzymes [69,71,192,193]. When taken together, these studies highlight the importance of normal iron content to the heart and that its deficiency could play a causal role in the pathogenesis of systolic and diastolic myocardial dysfunction as well as HF progression independently of systemic iron status.

Replenishing iron prevents abnormalities of Ca^2+^ handling, improves cardiac function and survival in rat models with HF [180]. In the Myocardial-IRON Trial, it was found that administering FCM intravenously in iron-deficient HF patients resulted in significant improvement in cardiac magnetic resonance sequences, indicating myocardial iron repletion [220]. This correction of ID was accompanied by improved right and left ventricular ejection fraction on the 7th day already [221]. Several other studies corroborate these positive echocardiographic effects of IV on the heart [216,222,223,224,225]. Remarkably, in HFrEF patients receiving cardiac resynchronisation therapy (CRT), the presence of ID is associated with diminished reverse remodelling and lesser likelihood of functional improvement after CRT implementation, suggesting that adequate myocardial iron content is a prerequisite to derive optimal benefits from CRT implantation with respect to reverse cardiac remodelling and improved cardiac function [226,227]. In line with these findings, the recent IRON-CRT trial showed that IV iron repletion reverses myocardial remodelling and boosts cardiac performance and contractility in patients receiving CRT, which were also accompanied by improvements in quality of life and exercise capacity [62]. These results demonstrate the incremental potential of IV iron in reverse cardiac remodelling in addition to guideline-directed therapies that induce reverse remodelling [228]. Whether IV iron can prevent myocardial remodelling inflicted by either ischaemic or non-ischaemic damage is not known. It is worth noting that although beneficial effects of IV iron on the failing heart are indubitable, some research indicates that mitochondrial iron chelation might also have beneficial effects for patients with HF [177,218,229,230]. Further work is needed to delineate the effects of IV iron both systemically and cellularly.

### 3.3. Skeletal Muscles

Exercise intolerance is a cardinal symptom of HF, with impaired oxidative metabolism, decreased blood perfusion to skeletal muscles and oxygen delivery implicated as potential causes [231]. Mitochondrial dysfunction inflicted by ID is not limited to the heart but extends to other organs, especially those with high energy demands such as skeletal muscles. Intuitively, decreased exercise capacity in ID is linked to defective O2 delivery due to anaemia. However, deficiency of iron impairs skeletal muscle function also by anaemia-independent pathways, which is oxidative metabolism and oxygen storage in myoglobin [43,232]. Several animal studies have shown that impaired exercise capacity is directly linked to ID due to diminished mitochondrial energy metabolism [43,233]. Even when hemoglobin levels were kept constant, iron-deficient animals showed a significantly lower exercise capacity accompanied by impaired oxidative metabolism, indicating a direct relationship between ID and impaired physical performance irrespective of anaemia [234,235]. A meta-analysis in athletes with isolated ID without anaemia showed that iron therapy improves systemic iron status and their aerobic capacity as evaluated using maximal oxygen consumption (VO2 max) [236]. Furthermore, in a knockout mice model of Tfr1 specifically to skeletal muscles, Barrientos et al. showed that isolated muscle ID had profound systemic metabolic effects besides impaired mitochondrial respiration in muscles, suggesting that muscle ID may have unrecognised effects on systemic energy homeostasis [237].

In patients with HF, results of the FAIR-HF, CONFIRM-HF and EFFECT-HF randomised controlled trials all indicate that iron repletion improved exercise capacity irrespective of attained hemoglobin levels [22,23,24]. Melenovsky et al. showed that iron-deficient HF patients exhibit more severe skeletal muscle myopathy than their iron-sufficient counterparts as assessed using Phosphorus-31 magnetic resonance spectroscopy (^31^P MRS). By using the same approach, Charles-Edwards et al. showed that in iron-deficient HFrEF, IV iron isomaltoside significantly shortens phosphocreatine regeneration after exercise despite no change in haemoglobin levels, indicating enhanced skeletal muscle energetics already by 2 weeks [238]. Boosting skeletal muscle energetics seems to be an important mechanism by which correcting ID leads to beneficial outcomes in patients with HF.

### 3.4. Kidneys

The kidneys are another high-energy-demanding organ system with high mitochondrial content [239]. From an energetics perspective, reabsorption and secretion of solutes in the nephrons occur either passively or actively (e.g., Na^+^/K^+^-ATPase), with the latter requiring energy for proper functioning. As such, iron, due to its crucial role in the mitochondria, is important for kidney function [240]. Although ID is mostly seen as a consequence of renal diseases, several studies suggest that iron itself influences kidney function [241]. A recent Mendelian randomisation study investigated the causal effect of serum iron levels on kidney function in the general population. They found a 1.3% increase in estimated glomerular filtration rate per standard deviation increase in serum iron (95% confidence interval 0.4–2.1%; *p* = 0.004), indicating a protective effect of higher iron levels on renal function [242]. In accordance with these findings, feeding rats an iron-deficient diet resulted in an increase in malondialdehyde (an indicator of increased lipid peroxidation) in the kidneys, suggesting that ID can also adversely affect the kidney through oxidative stress and mitochondrial dysfunction [210]. In children, ID was shown to be associated with tubular and glomerular damage accompanied by increased oxidative stress markers [243]. Whether ID has similar direct effects on the kidneys in patients with HF is unknown.

In patients with HF, Toblli et al. showed that IV iron in anaemic HF patients with CKD resulted in a significant improvement in renal function [244]. Similarly, in a sub-analysis of the FAIR-HF trial, it was found that patients in the FCM group had an improved kidney function as evaluated by estimated Glomerular Filtration Rate (eGFR) [245]. This improvement in renal function was observed in all pre-specified subgroups, including HF patients with preserved renal function and those without anaemia. Remarkably, correcting ID might be associated with a reduction in fibroblast growth factor 23 (FGF23) [246,247], which was linked with worse clinical outcomes in both HF and renal disease [248]. This suggests that the benefits of IV iron on the kidneys may extend beyond its effects on the energetics of renal mitochondria. Future studies should look into the interaction between FGF23, iron status, heart and kidneys since FGF23 has been shown to decrease hepcidin expression [249], in addition to acting as a mediator between ID and its association with mortality in patients with HF [250].

Altogether, treating ID in HF patients may pose renoprotection properties. Data on the effects of ID on the kidneys are scarce. Further studies are therefore warranted. Moreover, given the established role of the kidneys in controlling systemic iron levels [241], it is yet to be ascertained whether impaired kidney function could increase urinary iron excretion and thereby cause ID in HF.

### 3.5. The Immune System

Beyond the cardiocentric perspective, iron homeostasis is also important for the immune system [43]. The relationship between iron and immunity is complex and bidirectional. As alluded to earlier, inflammation as an immune effector mechanism can lead to iron dysmetabolism, but iron dysmetabolism itself also causes adverse changes in immune function [129,251].

ID can affect the immune system in multiple ways. ID negatively affects both the growth and effector mechanisms of the immune system. Deleterious effects of ID include reduced neutrophil activity (myeloperoxidase is iron-dependent), reducing functions of nuclear factor kappa and HIFs, nitric oxide (NO) formation, a defective proliferation of T cells (especially T helper 1 cells) [252] and impaired interleukin 2 production [44,253,254]. Howden et al. showed that activated CD4+ and CD8+ T-cells upregulate their transferrin receptor, suggesting that iron is important for T-cell activation [255]. In mice fed an iron-deficient diet, altered T lymphocyte and natural killer (NK) cell activity was demonstrated, indicating impaired cellular mediated immunity [256,257].

Contrary to the overly simplistic view where ID is merely seen as a consequence of inflammation, studies showed that ID could perpetuate and amplify inflammation [127]. ID in mice induces and enhances inflammation when compared to mice with normal iron status [258]. A recent study in a rat post-myocardial infarction (MI) HF model showed that iron supplementation (FCM) reduces inflammation [208]. This dampening effect of iron is thought to be due to the important role of iron for immune cells to mount an effective immune response. In fact, more than 10 years ago, Toblli et al. showed that administration of iron sucrose intravenously in anemic HF patients led to a significant reduction in C-reactive protein [244]. Other studies corroborated this evidence [207]. When taken together, these results support the idea that ID itself can also have a direct adverse effect on the immune system and that its correction has advantages beyond merely stimulating erythropoiesis.

### 3.6. The Brain

Mental functions have biochemical bases, and hence dysregulation herein can lead to mental effects. A growing body of evidence suggests that iron is important for neurological functions as the brain is also a metabolically active organ, making it particularly susceptible to ID [259]. In addition to energy deficits of neural cells, ID can also impair synaptic plasticity, myelination and reduce the activity of multiple iron-dependent enzymes involved in dopamine and serotonin synthesis (monoamine oxidase, tyrosine hydroxylase and tryptophan hydroxylase) [260,261]. Several studies showed that brain ID leads to deficits in memory, learning, behaviour and emotional problems [262]. Low levels of serotonin due to ID may lead to a relapse of depression [263]. Psychological disorders such as depression are very common in HF patients [264]. Several studies reported a higher prevalence of depression in iron-deficient HF [26,265,266]. Whether systemic ID is associated with brain ID in HF patients is unknown. Moreover, the effects of IV iron in patients with HF on mental functioning have not been assessed yet.

### 3.7. Thyroid Gland

Accumulated evidence shows that thyroid dysfunction is linked with an increased risk for and worsening of HF [8,267,268]. ID impairs thyroid hormone metabolism by different mechanisms, including ineffective erythropoiesis, reduced thyroid peroxidase activity (haeme-containing enzyme) and increased hepatic inactivation of thyroid hormones [269,270]. Iron deficient HFpEF patients have a significantly higher prevalence of thyroid disease [265]. The therapeutic consequence of replenishing iron on thyroid function in HF has not been studied yet.

## 4. Novel Therapeutic Options for Targeting Iron Metabolism

Although iron replacement therapy remains to be the cornerstone of treating ID in HF, several experimental iron metabolism-targeting agents have been developed to treat IDA, many of which were evaluated in patients with CKD and cancer-related anaemia [271]. These treatment options increase iron absorption as well as the mobilisation of sequestered iron by either downregulating the synthesis and/or function of hepcidin or by stabilising HIFs as a result of Prolyl-4-hydroxylases (PHDs) inhibition [249].

Manipulation of the hepcidin–ferroportin axis seems an attractive target as the interplay between hepcidin and ferroportin is crucial for regulating iron status and aberrations in this pathway are centrally involved in the pathophysiology of FID [9]. Targeting hepcidin directly (e.g., LY2787106, Lexaptepid pegol or Anticalins), or indirectly by targeting inflammatory markers (e.g., IL-6), bone morphogenic protein 6 (BMP6) [LY3113593], was proposed as a treatment option for anaemia of inflammation as these agents were shown to increase intestinal iron absorption and mobilisation in phase 1 and/or 2 studies [249]. Moreover, blocking hepcidin’s interaction with ferroportin using ferroportin antibodies (LY2928057) reduces ferroportin internalisation, thereby increasing mobilisation of sequestered iron from the reticuloendothelial system [272]. Manipulation of the hepcidin pathway in inflammatory conditions such as HF might be a compelling option to treat ID in HF besides targeting inflammation, especially in those with FID as they have clear inflammatory components accompanied by high hepcidin levels [71]. In a recent phase 1/2 clinical trial, Pergola et al. showed that administration of ziltivekimab, anti-IL-6 ligand antibody, led to a dose-dependent improvement in serum iron, TSAT, inflammatory markers and reduction in hepcidin in CKD patients on haemodialysis with hyporesponsiveness to erythropoiesis-stimulating agents [123]. Whether targeting inflammation by reducing IL-6 activity and thereby improving clinical outcomes and iron metabolism can also be a therapeutic option to patients with HF is unknown.

Another promising approach is the manipulation of the prolyl hydroxylase domain/hypoxia-inducible factor (PHD/HIF) pathway, which seems to be deranged in iron-deficient patients with HF [12,185]. Several HIFs stabilisers are being developed, some of which have entered/finished phase 3 of clinical trials, including Vadadustat, Daprodustat and Roxadustat [273]. Meta-analyses of several randomised controlled trials showed that PHD inhibitors increase the levels of hemoglobin, serum transferrin and increase intestinal iron absorption while reducing levels of hepcidin in anaemic CKD patients [273,274,275]. These effects seem to be independent of inflammation [273]. Such activation of HIF signalling can provide a physiologic approach towards improved iron metabolism and may complement and/or reduce the need for IV iron in iron-deficient patients with HF. Besides being orally administered, targeting this pathway may have other non-erythopoetic benefits, such as lowering cholesterol levels as well as blood pressure [276,277]. However, there are many concerns regarding its safety as stabilising HIFs might lead to unwanted effects such as promoting cancer development since HIFs modulate the expression of various proteins that are involved in energy metabolism, angiogenesis, cellular growth and differentiation [249]. Large, carefully designed, long-term clinical trials are required to clearly understand the effects of systemic activation of the HIF pathway on iron-deficient HF patients.

Lastly, it should be noted that these agents can mainly increase absorption or mobilisation of sequestered iron, making them potentially ineffective for patients who have AID. Whether targeting the PHD/HIF or hepcidin–ferroportin axes have additive or possibly even synergistic effects with IV iron to correct ID, improve iron mobilisation in a more physiological manner and prevent relapses of ID remains to be investigated.

## 5. Current Knowledge Gaps

**Figure 4:**
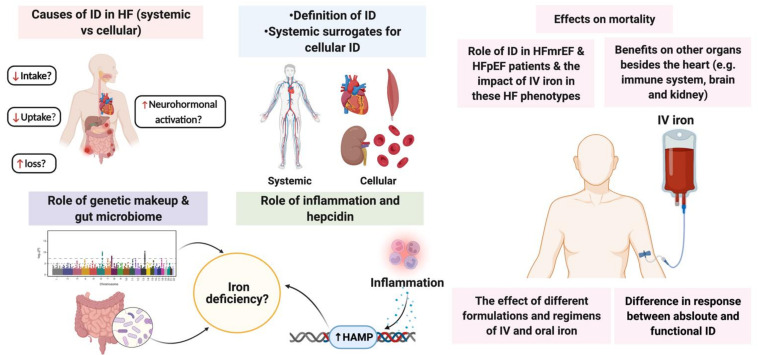
Summary of current knowledge gaps and future directions regarding iron deficiency in heart failure. ID: iron deficiency; HAMP: hepcidin; IV: intravenous; HF: heart failure; HFmrEF: heart failure with mildly reduced ejection fraction; HFpEF: heart failure with preserved ejection fraction. (Created with BioRender.com, accessed on 24 November 2021).

In this review, we summarised the current understanding of ID pathophysiology as well as the molecular basis potentially explaining improvements observed in patients with HF after administration of IV iron. Despite more research being dedicated to understanding the role of ID in HF, countless aspects remain unresolved. Firstly, current mechanistic studies trying to explain the benefits of IV iron are cardiocentric in nature, while the role of iron is not limited to the heart. The studies observing the benefits of IV iron on other organs, e.g., the brain and kidneys, will facilitate understanding the pathophysiology of ID. Secondly, thus far, trials have considered patients with AID and FID as one homogeneous group, while observational studies show different associations between FID and AID [71,278]. Whether these two groups are actually heterogeneous and might respond differently to IV iron is still not completely understood. Thirdly, current evidence about IV iron in HF is mainly derived from trials using FCM, and minimal investigation has been performed on non-FCM iron supplements despite past and ongoing trials using other preparations (e.g., isomaltoside iron). There are limited data comparing the efficacy and safety of the different preparations of IV iron as well as regimens (e.g., when and how much should be administered) in patients with HF. Fourthly, given the role of the microbiome in the pathophysiology of HF [119], the intricate relationship between iron and gut microbiome [279] and that oral iron treatment alters the composition of the gut microbiome [280,281], it is unknown whether the composition of the gut microbiome might play a role in causing ID in HF and whether iron replacement might confer favorable effects on the gut bacterial diversity. Another avenue of research is the fact that current knowledge of ID in HF is mainly from studies including HF patients with reduced LVEF, while the role of ID in preserved and mildly reduced LVEF remains equivocal. Ongoing studies will shed more light on the role of IDunknown whether the composition of the gut microbiome might in the different shades of HF, as well as whether correcting ID can improve the survival of HF patients. A summary of the current knowledge gaps discussed in this review is outlined in Figure 4.

## 6. Conclusions

The consequences of ID per se reach far beyond those of anaemia. Energy deficit is one of the hallmarks of HF. Mounting evidence indicates that mitochondrial dysfunction is a prominent repercussion of ID, which further aggravates the existing deficit in energy in HF. Current standard-of-care pharmacological approaches to HF provide symptomatic and clinical benefits by reducing the workload on the heart instead of increasing its reserve. Targeting ID in HF can improve the care of HF patients as it addresses this shortcoming by augmenting mitochondrial function. This makes ID stand out as a therapeutic target in HF since it is relatively easy to diagnose and treat with potential anti-remodelling effects.

Despite recognition of ID as a novel therapeutic target in HF, its origin remains unclear, with recent studies distinguishing between mechanisms leading to systemic and MID as changes in these compartments seem to be not parallel. Neurohormonal activation links myocardial iron content with the pathophysiological cascade of HF. Whether MID may be part of the cause of systemic ID or a consequence is unknown. Several factors make HF patients particularly a vulnerable population to develop systemic ID. Longitudinal, mechanistic and intervention studies are needed to investigate the contribution of the different factors implicated in the pathophysiology of ID in HF.

## Figures and Tables

**Table 1:** Overview of proteins that require iron to function properly.

Function	Protein
Oxygen transport	Hemoglobin
Oxygen storage	Myoglobin
Lipid and cholesterol biosynthesis	NADPH-cytochrome P450 reductase, fatty acid desaturases, cytochrome P-450 subfamily 51 and Cytochrome P450 Family 7 Subfamily A Member 1
Oxygen sensing and regulation of hypoxia	Hypoxia-inducible factor prolyl hydroxylases
Synthesis catecholamines and neurotransmitters	Tryptophan hydroxylase, tyrosine hydroxylase, monoamine oxidase and aldehyde oxidase
Host defence, inflammation and production of nitric oxide	Myeloperoxidase, NADPH oxidase, indoleamine 2,3- dioxygenase, nitric oxide synthase and lipoxygenases
DNA synthesis, replication and repair	Ribonucleotide reductases, DNA polymerases, DNA glycolsylases, DNA primases, DNA helicasess and DNA endonucleases. Dihydropyrimidine dehydrogenas
Collagen synthesis	Proline hydroxylase
Electron transport and respiratory chain	Cytochrome C oxidase, Cytochrome b, cytochrome c1, Cytochrome oxidase P540, NADH dehydrogenase, aconitase, citrate synthase, Succinyl dehydogease, cytochrome reductase, Complex I-III, rieske protein, NADH ferrocyanide oxidoreductase
Adrenoxin	Steroid hydoxylation
Antioxidant defence	Catalase
Response to oxidative stress	Glutathione peroxidase 2, lactoperoxidase
Amino acid metabolism	Tryptophan pyrrolase, Phenaylalanine hydroxylase, deoxyhypusine hydroxylase
Carnitine biosynthesis	α-ketoglutarate (αKG)-dependent oxygenases
Synthesis of thyroid hormone	Thyroid peroxidase
Drug detoxification	Cytochrome P450 , NADPH cytochrome P450 reductase
Prostaglandin thromboxane synthesis, inflammation and response to oxidative stress	Cyclooxyenase
microRNA biogenesis	DiGeorge Syndrome Critical Region Gene 8
Ribosome function and tRNA modification	ABCE1, CDKRAP1, TYW1 and CDKAL1, Methylthiotransferase
Haeme biosynthesis	Ferrochelatase
Apoptosis and oxygen transport in the brain	Neuroglobin
Purine metabolism and synthesis	Xanthine oxidase, amidophosphoribosyltransferase
